# Dexmedetomidine pretreatment alleviates cerebral ischemia/reperfusion injury by inhibiting neuroinflammation through the JAK2/STAT3 pathway

**DOI:** 10.1590/1414-431X2022e12145

**Published:** 2022-07-13

**Authors:** Huan Liu, Jianli Li, Li Jiang, Jinhua He, Huanhuan Zhang, Keyan Wang

**Affiliations:** 1 Department of Anesthesiology, Hebei General Hospital, Shijiazhuang, Hebei, China; 2 College of Postgraduate, Hebei North University, Zhangjiakou, Hebei, China

**Keywords:** Dexmedetomidine, Cerebral ischemia/reperfusion injury, Neuroprotection, JAK2/STAT3 signaling pathway, Inflammation

## Abstract

Dexmedetomidine (DEX) is known to provide neuroprotection against cerebral ischemia and reperfusion injury (CIRI), but the exact mechanisms remain unclear. This study was conducted to investigate whether DEX pretreatment conferred neuroprotection against CIRI by inhibiting neuroinflammation through the JAK2/STAT3 signaling pathway. Middle cerebral artery occlusion (MCAO) was performed to establish a cerebral ischemia/reperfusion (I/R) model. Specific-pathogen-free male Sprague-Dawley rats were randomly divided into Sham, I/R, DEX, DEX+IL-6, and AG490 (a selective inhibitor of JAK2) groups. The Longa score, TTC staining, and HE staining were used to evaluate brain damage. ELISA was used to exam levels of TNF-α. Western blotting was used to assess the levels of JAK2, phosphorylated-JAK2 (p-JAK2), STAT3, and phosphorylated-STAT3 (p-STAT3). Our results suggested that both pretreatment with DEX and AG490 decreased the Longa score and cerebral infarct areas following cerebral I/R. After treatment with IL-6, the effects of DEX on abrogating these pathological changes were reduced. HE staining revealed that I/R-induced neuronal pathological changes were attenuated by DEX application, consistent with the AG490 group. However, these effects of DEX were abolished by IL-6. Furthermore, TNF-α levels were significantly increased in the I/R group, accompanied by an increase in the levels of the p-JAK2 and p-STAT3. DEX and AG490 pretreatment down-regulated the expressions of TNF-α, p-JAK2, and p-STAT3. In contrast, the down-regulation of TNF-α, p-JAK2, and p-STAT3 induced by DEX was reversed by IL-6. Collectively, our results indicated that DEX pretreatment conferred neuroprotection against CIRI by inhibiting neuroinflammation via negatively regulating the JAK2/STAT3 signaling pathway.

## Introduction

Ischemic stroke is one of the most harmful neurological diseases and can cause irreversible brain injury with high teratogenicity and mortality. Although the restoration of blood flow is critical for promoting functional recovery of tissue in the ischemic areas, it may cause secondary damage, defined as cerebral ischemia/reperfusion injury (CIRI) (1). Clinically, acute CIRI may occur during the shock, cardiac arrest, or perioperative period for neurosurgery and cardiovascular surgery, especially in patients with poor physiological basis. Owing to anesthesia, operation, and other factors, perioperative I/R-induced brain injury is often difficult to be monitored in time and leads to poor prognosis (2). Therefore, exploring novel strategies to reduce or prevent I/R-induced brain injury during the perioperative period is still a major medical challenge.

Many anesthetics, such as isoflurane, sevoflurane, and ketamine, have been widely used to investigate neuroprotection and neurotoxicity and have been shown to have a contradictory effect on CIRI, thus the protective role of anesthetics in CIRI requires further investigation (3,4). Dexmedetomidine (DEX), a potent α2-adrenergic receptor agonist, is known for its sedative, analgesic, anti-sympathetic, and anti-anxiety effects and is widely used during the perioperative period (5). Accumulating evidence has shown that DEX can alleviate I/R injury in a variety of organs, including the kidney, heart, lung, spinal cord, and intestine, and its neuroprotective effect against I/R-induced brain injury has also been widely studied (6). Although there have been many reports on the neuroprotective effects of DEX, the exact molecular mechanism underlying these effects has not been determined. One possible mechanism of its neuroprotection is through its anti-inflammatory effects, and studies have found that DEX exerts neuroprotection against CIRI by inhibiting the expressions of pro-inflammatory cytokines, such as tumor necrosis factor (TNF)-α, interleukin (IL)-6, and IL-1β (7). A previous study showed that the α2-adrenoreceptor antagonist yohimbine inhibited the anti-inflammatory effect of DEX and eliminated the neuroprotective effect of DEX following cerebral I/R (8). Although activation of the α2-adrenoreceptor pathway has been identified as one of the mechanisms by which DEX suppresses inflammation and exerts neuroprotection against CIRI, the precise mechanism remains obscure.

As one of the important inflammatory factors, IL-6 promotes the aggregation of neutrophils and the release of inflammatory factors by activating the JAK/STAT pathway through binding to the IL-6 receptor, which aggravates nerve damage (9,10). The JAK/STAT pathway consists of two families of proteins, JAKs (JAK1, JAK2, JAK3, and TYK2) and STATs (STAT1, STAT2, STAT3, STAT4, STAT5a, STAT5b, and STAT6), and participates in regulating the expression of genes related to proliferation, differentiation, immunity, and apoptosis (11). JAK2 and STAT3 are considered to be the most-conserved and most-ancient members of the JAK/STAT pathway and play an important role in multiple pathological regulatory processes in the CNS, such as Alzheimer's disease, Parkinson's disease, and cerebral ischemia diseases (12-14). A previous study has confirmed that the expression of p-JAK2 and p-STAT3 were up-regulated after CIRI, ultimately resulting in massive release of inflammatory factors, while AG490, an inhibitor of JAK2, and STAT3 siRNA exerted significant neuroprotection and contributed to neurological recovery (14). The JAK2/STAT3 pathway is inactivated in most cases, and once excessively activated, may be harmful to neuronal growth and normal function. Moreover, several *in vivo* studies confirmed that DEX exerts an organ-protective effect against I/R injury by down-regulating the levels of p-JAK2 and p-STAT3 proteins (15,16). Recently, an *in vitro* study demonstrated that DEX might inhibit the activation of the JAK/STAT signaling pathway and decrease oxygen-glucose deprivation-induced astrocyte apoptosis (17). Additionally, a study reported that DEX provided brain protection in the process of cardiopulmonary bypass by reducing the inflammatory response through regulating the JAK2/STAT3 signaling pathway negatively (18). The above studies indicated that the mechanism underlying the protective role of DEX in I/R injury might be related to the JAK2/STAT3 signaling pathway. However, whether DEX can inhibit neuroinflammation in response to CIRI in rats via the JAK2/STAT3 signaling pathway remains unknown.

Based on the above evidence, we established a cerebral I/R rat model to investigate whether DEX alleviated CIRI by inhibiting neuroinflammation through the JAK2/STAT3 pathway.

## Material and Methods

### Animals

A total of 125 specific-pathogen free male Sprague-Dawley rats (8-10 weeks old, 280-330 g) were purchased from the Experimental Animal Center of Hebei Medical University (China). Rats were housed in a standard room at constant temperature (23±2°C) and suitable humidity (55±5%), under a 12-h light/dark cycle. All animals received a standard diet and had free access to food and water. This experiment was started after the rats had adapted to the new environment for at least one week. All experiments were performed in accordance with The National Institutes of Health Guide for the Care and Use of Laboratory Animals, and this study was approved by the Animal Ethics Committee of Hebei General Hospital.

### Establishment of the animal model

The transient focal cerebral I/R model was established by middle cerebral artery occlusion (MCAO) for 2 h followed by 24-h reperfusion as described in a previous study (19). Rats were anesthetized by intraperitoneal injection of 3% sodium pentobarbital (50 mg/kg) and fixed to the operating table in a supine position. Then, a midline incision was made to separate the left common carotid artery (CCA), the external carotid artery (ECA), and the internal carotid artery (ICA). A small V-shaped incision was made at the fork of the left CCA using ophthalmic scissors after ligation of the CCA and ECA and clamping of the ICA. A silicon-coated nylon monofilament (diameter 0.38±0.02 mm, Beijing Cinontech Co. Ltd., China) was inserted into the ICA through this small V-shaped incision and then slowly pushed and stopped when blocked, which indicated that the monofilament has reached the origin of the MCA. After 2 h of MCAO, the monofilament was withdrawn to allow reperfusion. All the procedures were performed without the monofilament insertion in the Sham group.

### Experimental protocols

**Figure 1:**
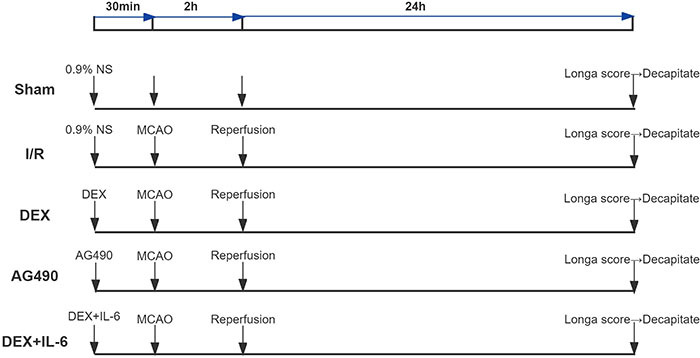
Experimental groups and protocols. MCAO: middle cerebral artery occlusion; I/R: ischemia/reperfusion; DEX: dexmedetomidine; NS: normal saline; IL-6: interleukin-6.

Rats (n=125) were randomly divided into 5 groups (n=25 per group): Sham, I/R, DEX, DEX+IL-6 (a major activator of JAK/STAT), and AG490 (a selective inhibitor of JAK2) (Figure 1). Specifically, the DEX group was treated with intraperitoneal injection of DEX (50 µg/kg, Yangtze River Pharmaceutical Group, China) 30 min before ischemia. The I/R group was operated to induce CIRI and received an intraperitoneal injection of an equal amount of saline. The Sham group received an intraperitoneal injection of an equal amount of saline without I/R operation. The DEX+IL-6 group received an I/R operation with the intrathecal IL-6 administration (1 µg, CLOUD-CLONE CORP., China) and intraperitoneal injection of DEX (50 µg/kg) 30 min before ischemia. In addition, the AG490 group rats were treated with an intraperitoneal injection of AG490 (10 mg/kg, MCE LLC, China) 30 min before ischemia.

### Evaluation of neurological function

The Longa score was used to evaluate neurological deficits as previously reported (19) and recorded 24 h after reperfusion. The scores were: 0 points: normal, no neurologic deficit; 1 point: mild neurological deficit, the forelimb contralateral to the lesion could not be fully extended when pulling the tail; 2 points: moderate neurological deficit, turning to the opposite side of the lesion while walking; 3 points: severe neurologic deficit, falling to the contralateral side of the lesion when walking; and 4 points: could not spontaneously walk and had poor consciousness. Neurological symptoms were scored using a single-blind method.

### TTC staining

After neurological function assessment, 5 rats from each group were sacrificed. Subsequently, the brains were quickly removed and placed in a −20°C freezer for 20 min after removal of the olfactory bulb and cerebellum. Frozen rat brains were cut into 5 pieces on average along the optic chiasma backward, and each slice was approximately 2-mm thick. The pieces were stained with 2% TTC (2,3,5-triphenyltetrazolium chloride; Beijing Solarbio Science & Technology Co., Ltd., China) solution at 37°C under dark for 30 min, then fixed in 4% paraformaldehyde for 24 h, and photographed. Images were processed with Image-Pro Plus software (Media Cybernetics Inc., USA), and the degree of cerebral infarction is reported as the ratio between the infarct area and the entire brain area.

### HE staining

After neurologic assessment, 5 rats from each group were sacrificed for HE staining. The brain tissue of the rats was immediately removed and immersed overnight in 4% paraformaldehyde solution. Brain tissue sections were cut approximately 4-μm thick after the brain tissue was dehydrated and embedded in paraffin. After routine dewaxing and hydration, these sections were placed in hematoxylin for 5 min and stained with eosin for 3 min. The pathological changes of neurons in the cerebral cortex were observed under an optical microscope (ECLIPSE Ni-U, Nikon, Japan). The number of normal neurons was calculated in three different fields, and the calculation was repeated three times per sample.

### ELISA

After neurologic assessment, 5 rats/group were sacrificed and the levels of TNF-α in brain cortex tissue were detected by ELISA. Cortex tissue was homogenized in PBS and centrifuged at 9600 *g* for 5 min at 4°C, and the protein concentrations of the supernatant were detected by BCA assay. Then, TNF-α levels were measured by specific ELISA kits (Cloud-Clone Corp., China) according to the manufacturer's instructions. Briefly, the ELISA kit was kept in equilibrium for 20 min at room temperature, and 100 μL supernatant was incubated on an ELISA plate at 37°C for 60 min. Then, 100 μL Detection A solution was added to the ELISA plate and incubated at 37°C for 30 min, then 100 μL Detection B solution was added for incubation at 37°C for 30 min. After the addition of 90 μL TMB substrate solution (3,3',5,5'-tetramethylbenzidine) and 50 μL stop solution, protein levels were determined according to the absorbance at 450 nm.

### Western blot

After neurological function assessment, 5 rats in each group were sacrificed and characteristic proteins in brain cortex tissue were detected by Western blot. Cortex tissues were homogenized in lysis buffer, and total protein was extracted after centrifugation (13,800 *g* for 15 min at 4°C). Then, the protein concentration was evaluated by BCA. The protein sample was separated in a 10% SDS-PAGE, and then transferred to PVDF membranes. Membranes were sealed in a TBST solution containing 5% skimmed milk powder at room temperature for 2 h and incubated with the following primary antibodies overnight at 4°C: anti-JAK2 antibody (1:1000, Abways, China), anti-p-JAK2 antibody (Tyr1007/1008) (1:500, ZEN-BIOSCIENCE, China), anti-STAT3 antibody (1:2000, Cell Signaling Technology, USA), anti-p-STAT3 antibody (Tyr705) (1:1000, Cell Signaling Technology), and anti-β-actin antibody (1:5000, Abways). Next, the membranes were incubated with an HRP-conjugated secondary antibody (1:10000, Abways) at room temperature for 1 h. An ECL kit (Beijing 4A Biotech Co., Ltd, China) was used to detect the protein bands, and the gray-scale quantification was performed by ImageJ (NIH, USA) software. β-actin served as an internal reference. Relative levels of the target protein are reported as the ratio of the gray value of this protein to the gray value of the β-actin protein.

### Statistical analysis

SPSS 26.0 statistical software (IBM, USA) was used to analyze data and the data are reported as means±SD. Among multiple groups, the data of normal distribution were analyzed by one-way analysis of variance (ANOVA), which was followed by the LSD test. The data of skew distribution are reported as median and interquartile range and were analyzed by a non-parametric Kruskal-Wallis test. A P value of <0.05 was considered to be a statistically significant result.

## Results

### DEX improved neurological deficits induced by cerebral I/R

**Figure 2:**
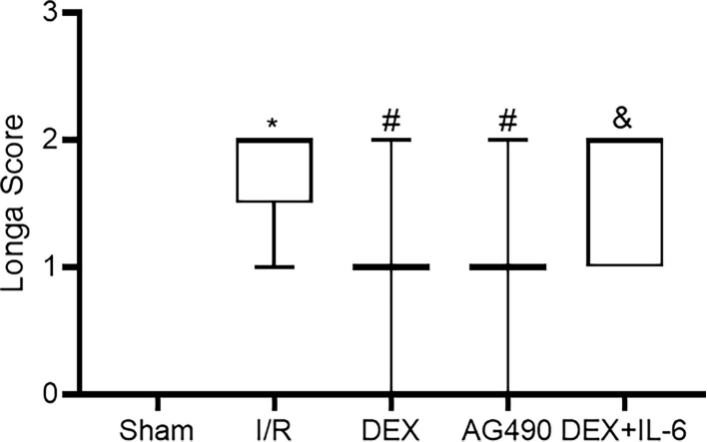
Neurological deficits were evaluated by the Longa score. Data are reported as median (interquartile range), n=25/group. *P<0.05 *vs* Sham group; ^#^P<0.05 *vs* I/R group; ^&^P<0.05 *vs* DEX group (nonparametric Kruskal-Wallis test). I/R: ischemia/reperfusion; DEX: dexmedetomidine; IL-6: interleukin-6.

Based on previous reports, 50 µg/kg DEX was selected as the appropriate concentration for our experiment (8,16). To investigate the effect of DEX on CIRI, we used Longa's five-grade standard scoring method to evaluate the neurological function of rats in each group. As shown in Figure 2, when compared with the Sham group (0.00 [0.00, 0.00]), the Longa score of the I/R group was markedly higher (2.00 [1.50, 2.00]). The Longa score in the DEX group (1.00 [1.00, 1.00]) was remarkably decreased compared with the I/R group, and the effect of DEX was abolished by intervention with IL-6 (2.00 [1.00, 2.00]). Pretreatment with AG490 significantly reduced the Longa score following CIRI (1.00 [1.00, 1.00]), consistent with the DEX group. These results suggested that DEX administration could provide neuroprotective effects against CIRI in rats.

### DEX decreased cerebral infarction area after CIRI

**Figure 3:**
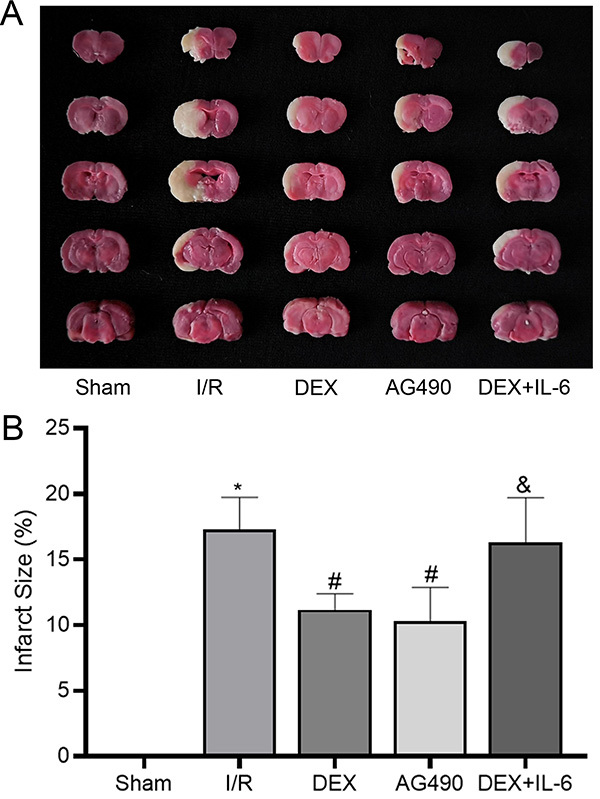
A, TTC staining of rat brain tissue. **B**, Percentage of cerebral infarction area. Data are reported as means±SD, n=5. *P<0.05 *vs* Sham group; ^#^P<0.05 *vs* I/R group; ^&^P<0.05 *vs* DEX group (one-way ANOVA). I/R: ischemia/reperfusion; DEX: dexmedetomidine; IL-6: interleukin-6.

As shown in Figure 3, the normal brain tissue was stained red and the infarcted tissue was white. Our results suggested that the infarct area increased after cerebral I/R (17.31±2.44%). While administration of DEX (11.18±1.20%) or AG490 (10.31±2.56%) reduced the cerebral infarction area following CIRI, no significant differences were observed between them. Compared with the DEX group, cerebral infarction area was significantly increased in the DEX+IL-6 group (16.32±3.39%). These results showed that DEX pretreatment could reduce the infarct area following cerebral I/R and confer neuroprotection.

### Effect of DEX on the histopathological changes induced by cerebral I/R

**Figure 4:**
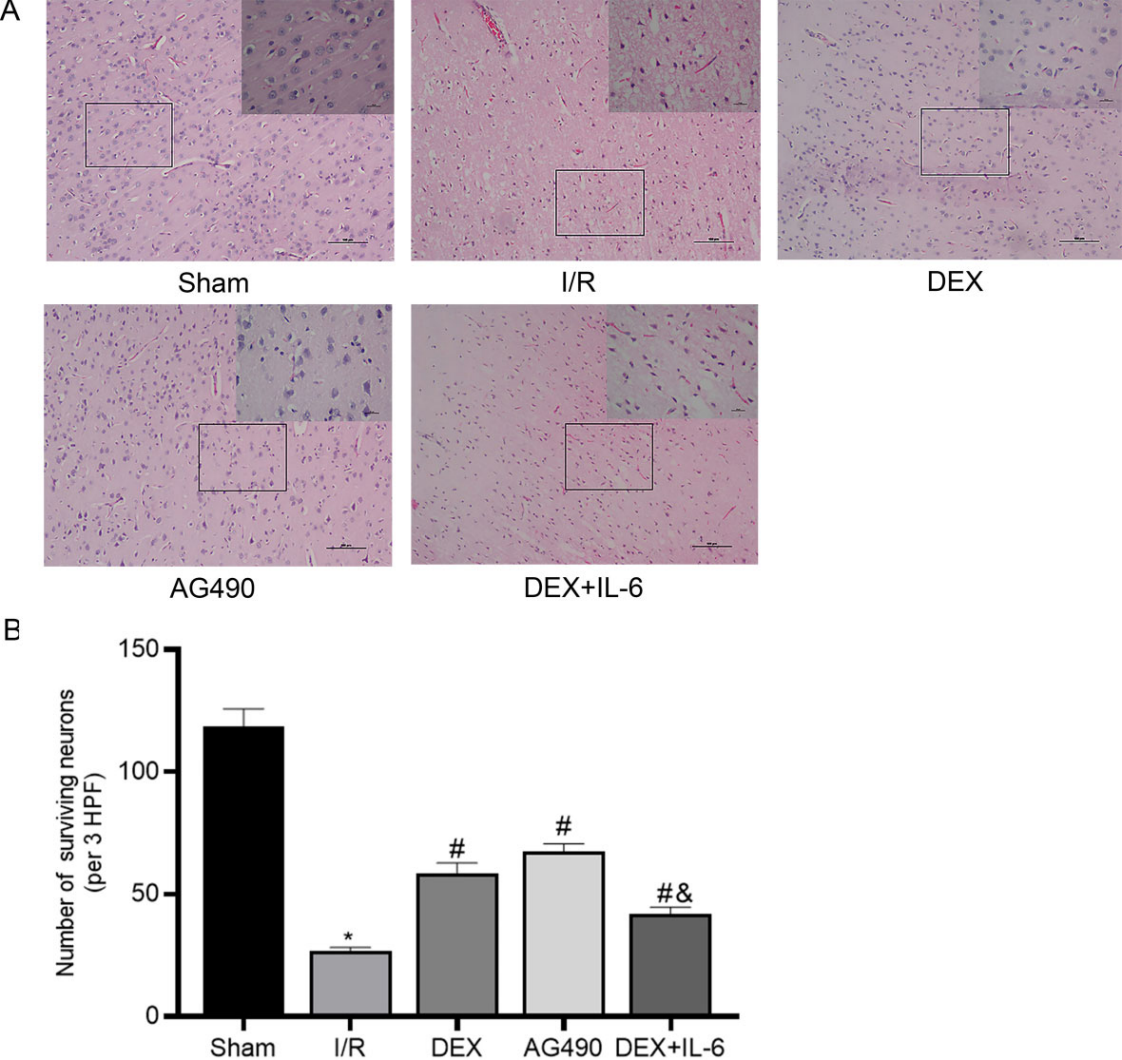
A, HE staining (×100 and 400, scale bars 100 and 20 µm) of rat brain tissue. **B**, Number of surviving neurons (per 3 HPF). Data are reported as means±SD, n=5. *P<0.05 *vs* Sham group; ^#^P<0.05 *vs* I/R group; ^&^P<0.05 *vs* DEX group (one-way ANOVA). I/R: ischemia/reperfusion; DEX: dexmedetomidine; IL-6: interleukin-6; HPF: high-power fields.

As shown in Figure 4, neurons in the cortex of the Sham group were orderly arranged, clearly outlined, and had complete morphology. Neurons in the I/R group showed obvious heterogeneity, and the number of surviving neurons in the I/R group (26.67±1.53) was notably decreased compared with the Sham group (118.33±7.37), which suggested that cerebral I/R could lead to large amounts of neuronal death. Pretreatment with DEX or AG490 could attenuate the neuron pathological changes following cerebral I/R, and the number of surviving neurons was significantly increased in the DEX (58.67±4.04) and AG490 groups (67.33±3.21). Furthermore, the degree of the pathological changes was slightly greater in the DEX+IL-6 group than in the DEX group, and the number of surviving neurons (42.00±2.65) was significantly reduced compared with the DEX group. These results further showed that DEX alleviated the neuronal damage and protected the neuronal integrity following cerebral I/R in rats.

### DEX inhibited release of the inflammatory factor TNF-α caused by cerebral I/R

**Figure 5:**
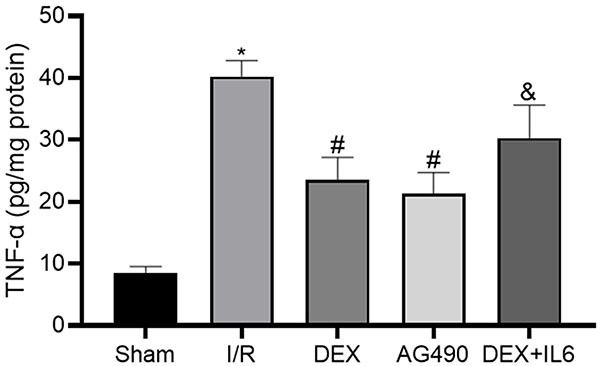
The level of tumor necrosis factor (TNF)-α was detected by ELISA. Data are reported as means±SD, n=5. *P<0.05 *vs* Sham group; ^#^P<0.05 *vs* I/R group; ^&^P<0.05 *vs* DEX group (one-way ANOVA). I/R: ischemia/reperfusion; DEX: dexmedetomidine; IL-6: interleukin-6.

Inflammatory response is one of the most important mechanisms involved in the process of CIRI (20). As shown in Figure 5, levels of TNF-α in the I/R group (40.22±2.57) were significantly increased compared to the Sham group (8.55±0.98). Compared to the I/R group, levels of TNF-α were significantly decreased in the DEX (23.47±3.73) and AG490 (21.25±3.49) groups, while there were no significant differences between the DEX and AG490 groups. However, levels of TNF-α in the DEX+IL-6 group (30.31±5.31) were increased compared with the DEX group. These results showed that DEX pretreatment could exert neuroprotective effects against CIRI through an anti-inflammatory mechanism.

### Effect of DEX on the levels of proteins related to the JAK2/STAT3 signaling pathway

**Figure 6:**
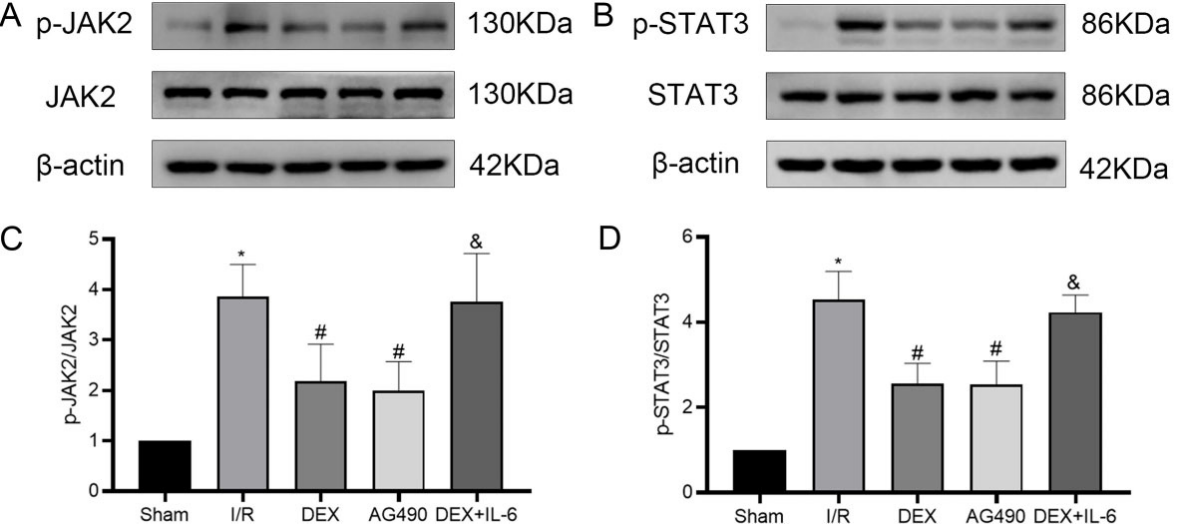
The protein levels of p-JAK2/JAK2 and p-STAT3/STAT3 were detected by western blotting. Representative western blots for **A**, JAK2 and p-JAK2, and **B**, STAT3 and p-STAT3. Densitometry analysis of western blots for the **C**, p-JAK2/JAK2 ratio, and **D**, p-STAT3/STAT3 ratio. Data are reported as means±SD, n=5. *P<0.05 *vs* Sham group; ^#^P<0.05 *vs* I/R group; ^&^P<0.05 *vs* DEX group (one-way ANOVA). I/R: ischemia/reperfusion; DEX: dexmedetomidine; IL-6: interleukin-6.

Activation of JAK2 leads to phosphorylation of STAT3 and allows it to enter the nucleus and regulate gene expression (11). Moreover, activation of the JAK/STAT pathway promoted the release of inflammatory factors and aggravated nerve injury (9,10). From the above results, we found that DEX could alleviate neuroinflammation induced by cerebral I/R, but the exact molecular mechanism remains unclear. To evaluate the precise mechanism underlying the neuroprotection of DEX against CIRI in rats, we used western blotting to assess the protein levels of the JAK2/STAT3 pathway in the brain cortex tissue. As shown in Figure 6, we observed that the ratios of p-JAK2/JAK2 and p-STAT3/STAT3 in the cortex tissue were significantly increased in the I/R group (3.86±0.63; 4.53±0.66). After pretreatment with DEX, the ratios of p-JAK2/JAK2 and p-STAT3/STAT3 were significantly decreased (2.18±0.73; 2.56±0.47), which were consistent with the AG490 group (1.99±0.58; 2.54±0.54). As expected, the protective effects of DEX were abolished by IL-6 (3.76±0.95; 4.23±0.40). Moreover, the Longa score, TTC staining, and HE staining also showed that the neuroprotective mechanism of DEX was related to the JAK2/STAT3 pathway. Taken together, these data suggested that DEX pretreatment attenuated CIRI by inhibiting neuroinflammation through the JAK2/STAT3 pathway.

## Discussion

This study for the first time proposed that DEX pretreatment alleviated CIRI by suppressing neuroinflammation through the JAK2/STAT3 pathway. Our results revealed a new therapeutic strategy for CIRI and provided a theoretical basis for DEX serving as a clinical drug to treat/prevent perioperative cerebral I/R-induced injury.

Ischemic stroke is a serious nervous system disease, which often results in death or long-term disability. Once it occurs, common clinical treatment aims to restore perfusion as soon as possible. However, the recovery of blood flow is often accompanied by the occurrence of CIRI, which can further exacerbate the cerebral damage. To date, multiple mechanisms have been suggested to be involved in the process of CIRI, including the inflammatory response, oxidative stress, calcium overload, cell autophagy, and others (20). Numerous drugs have been used for the treatment of CIRI, including certain necroptosis inhibitors, free radical scavengers, and NMDAR antagonists, but some of them have not been approved for clinical application or exhibit poor clinical efficacy (21). Therefore, searching for appropriate clinical drugs to treat/prevent CIRI remains a major medical challenge.

DEX, as a sedative agent, has been widely used in clinical practice. Previous studies have shown that DEX exhibited neuroprotection in various neuronal injury models, such as traumatic brain injury, I/R-induced brain injury, anesthetic-induced neuronal injury, and neurodegeneration (22). In recent years, both *in vivo* and *in vitro* studies have shown that DEX can exert neuroprotective effects against CIRI (23-25). Despite its neuroprotective effects being a hot topic, the specific molecular mechanism underlying these effects on CIRI has not been fully clarified. Therefore, we established an MCAO rat model in the present study to imitate the process of CIRI to explore the neuroprotective effects of DEX and its related molecular mechanism. Our results of the Longa score, TTC staining, and HE staining supported that DEX could improve neurological deficits and histopathological changes, which were consistent with previous reports (7,23).

Inflammatory response is one of the crucial mechanisms involved in CIRI, and inhibition of inflammation is considered a target for the treatment of CIRI (26). Cytokines and chemokines released from the damaged tissue can promote the aggregation of leukocytes when acute cerebral ischemia occurs, generating a series of factors that aggravate tissue injury, such as reactive oxygen species, TNF-α, and IL-6 (27). The pro-inflammatory cytokine TNF-α is an essential regulator of neutrophil function, and increased levels are associated with the severity of CIRI. Previous studies have reported that DEX has anti-inflammatory effects on I/R injury in animal models, and its anti-inflammatory effects have also been observed in clinical trials (28). Therefore, it was reasonable to believe that DEX could provide anti-inflammatory effects against CIRI. In our study, we observed that DEX markedly reduced levels of TNF-α in brain cortex tissue after CIRI, indicating that DEX could reduce neuroinflammation and protect brain tissue from I/R-induced nerve damage.

In this study, we further investigated the molecular mechanisms underlying the neuroprotective effects of DEX against CIRI. The JAK/STAT pathway is an important intracellular signal-transduction pathway and has been proven to mediate various cellular activities, including immunity and inflammation. Previous studies have shown that the JAK2/STAT3 signaling pathway participates in the progression of CIRI (29). However, the role of JAK2/STAT3 signaling in the development of CIRI is controversial. Some studies have suggested that activation of the JAK2/STAT3 signaling pathway promotes apoptosis, angiogenesis, oxidative stress, and neuroinflammation, whereas other studies reached the opposite conclusion (29). At present, there have been many studies on the role of the JAK2/STAT3 pathway in CIRI, but whether the JAK2/STAT3 pathway participates in DEX-mediated reduction of the inflammatory response to prevent against CIRI has not been investigated. A previous study showed that DEX provided brain protection in rats that underwent cardiopulmonary bypass by reducing the protein expression of p-JAK2 and p-STAT3 (18). In addition, Feng et al. (17) reported that DEX inhibited oxygen-glucose deprivation-induced astrocyte apoptosis through down-regulating the levels of p-STAT1 and p-STAT3. In our study, we observed that administration of DEX caused a decrease in p-JAK2/JAK2 and p-STAT3/STAT3 protein ratios in brain cortex tissue of rats following CIRI. To further verify whether the protective effects of DEX were associated with the inhibition of the JAK2/STAT3 pathway, we used AG490 (a specific inhibitor of JAK2) and IL-6 (a major activator of JAK/STAT) to detect the levels of JAK2, p-JAK2, STAT3, p-STAT3, and TNF-α. Our results suggested that AG490 decreased the ratios of p-JAK2/JAK2 to p-STAT3/STAT3 and the level of TNF-α, and improved brain damage, which were consistent with DEX. In contrast, IL-6 reversed the neuroprotective effects of DEX on CIRI rats, which indicated that IL-6 may activate the JAK2/STAT3 pathway and aggravate brain damage. In addition, a previous study found that blocking IL-6 trans-signaling could exert protection against I/R-induced renal injury by suppressing STAT3 activation (30). Therefore, it could be speculated that DEX alleviated I/R-induced brain injury, and inhibition of the JAK2/STAT3 pathway was possibly mediated by decreasing IL-6 levels. Moreover, HE staining showed that IL-6 partially abolished the effects of DEX, which indicated that other pathways may contribute to the neuroprotective mechanism of DEX. Some previous studies have shown that DEX pretreatment could inhibit cerebral I/R-induced neuroinflammation via regulation of multiple signaling pathways, such as AMPK and TLR4/NF-kB (8,23).

In summary, the present results suggested that DEX pretreatment could ameliorate CIRI by inhibiting neuroinflammation through the JAK2/STAT3 pathway. Our results provide an experimental basis for the clinical application of DEX in the treatment/prevention of CIRI. However, further studies should consider the relationship between the neuroprotective effects of DEX with dose and administration time, and other potential mechanisms need to be explored in the future.
